# Metformin counteracts stimulatory effects induced by insulin in primary breast cancer cells

**DOI:** 10.1186/s12967-022-03463-y

**Published:** 2022-06-07

**Authors:** Domenica Scordamaglia, Francesca Cirillo, Marianna Talia, Maria Francesca Santolla, Damiano Cosimo Rigiracciolo, Lucia Muglia, Azzurra Zicarelli, Salvatore De Rosis, Francesca Giordano, Anna Maria Miglietta, Ernestina Marianna De Francesco, Veronica Vella, Antonino Belfiore, Rosamaria Lappano, Marcello Maggiolini

**Affiliations:** 1 Department of Pharmacy, Health and Nutritional Sciences, University of Calabria, 87036 Rende, Italy; 2 Breast Unit, Regional Hospital Cosenza, 87100 Cosenza, Italy; 3 Endocrinology, Department of Clinical and Experimental Medicine, University of Catania, Garibaldi-Nesima Hospital, 95122 Catania, Italy

**Keywords:** Metformin, Insulin, Insulin receptor, Breast cancer, BCAHC-1 cells

## Abstract

**Background:**

Metabolic disorders are associated with increased incidence, aggressive phenotype and poor outcome of breast cancer (BC) patients. For instance, hyperinsulinemia is an independent risk factor for BC and the insulin/insulin receptor (IR) axis is involved in BC growth and metastasis. Of note, the anti-diabetic metformin may be considered in comprehensive therapeutic approaches in BC on the basis of its antiproliferative effects obtained in diverse pre-clinical and clinical studies.

**Methods:**

Bioinformatics analysis were performed using the information provided by The Invasive Breast Cancer Cohort of The Cancer Genome Atlas (TCGA) project. The naturally immortalized BC cell line, named BCAHC-1, as well as cancer-associated fibroblasts (CAFs) derived from BC patients were used as model systems. In order to identify further mechanisms that characterize the anticancer action of metformin in BC, we performed gene expression and promoter studies as well as western blotting experiments. Moreover, cell cycle analysis, colony and spheroid formation, actin cytoskeleton reorganization, cell migration and matrigel drops evasion assays were carried out to provide novel insights on the anticancer properties of metformin.

**Results:**

We first assessed that elevated expression and activation of IR correlate with a worse prognostic outcome in estrogen receptor (ER)-positive BC. Thereafter, we established that metformin inhibits the insulin/IR-mediated activation of transduction pathways, gene changes and proliferative responses in BCAHC-1 cells. Then, we found that metformin interferes with the insulin-induced expression of the metastatic gene CXC chemokine receptor 4 (CXCR4), which we found to be associated with poor disease-free survival in BC patients exhibiting high levels of IR. Next, we ascertained that metformin prevents a motile phenotype of BCAHC-1 cells triggered by the paracrine liaison between tumor cells and CAFs upon insulin activated CXCL12/CXCR4 axis.

**Conclusions:**

Our findings provide novel mechanistic insights regarding the anti-proliferative and anti-migratory effects of metformin in both BC cells and important components of the tumor microenvironment like CAFs. Further investigations are warranted to corroborate the anticancer action of metformin on the tumor mass toward the assessment of more comprehensive strategies halting BC progression, in particular in patients exhibiting metabolic disorders and altered insulin/IR functions.

**Supplementary Information:**

The online version contains supplementary material available at 10.1186/s12967-022-03463-y.

## Background

Breast cancer (BC) represents the most diagnosed malignancy and leading cause of cancer death in women worldwide [1]. Despite many improvements gained in early detection and treatment of BC, the advanced disease still remains a main challenge [2–5]. Metabolic traits that are usually linked with type 2 diabetes (T2D), including hyperinsulinemia, dysglycemia, dyslipidemia, inflammation, oxidative stress and obesity, are well-known risk factors for BC and major contributors to its progression and metastatic dissemination [6, 7]. For instance, high plasma levels of insulin are associated with increased BC incidence and relapse, resistance to conventional and targeted therapies as well as poor outcome even in the absence of diabetes [8, 9]. Insulin regulates multiple signaling pathways implicated in the growth and metastatic features of BC by binding to and activating either the cognate receptor (IR) or the insulin-like growth factor 1 receptor (IGF-1R) [10–13]. In this regard, it should be mentioned that approximately 80% of BCs express high levels of IR and its autophosphorylation is associated with high BC mortality [14–16]. In particular, the isoform A of IR (IR-A) is frequently overexpressed in BC, where it regulates epithelial-to-mesenchymal transition (EMT), stem-like cell phenotype, cell invasion, metastasis and resistance to therapies [10, 17, 18]. Epidemiological and retrospective clinical data have demonstrated that a first-line drug for T2D, namely metformin, is associated with a low risk of BC and reduced cancer-related mortality rate in T2D patients [19–23]. Accordingly, extensive preclinical studies have indicated that metformin may exert antitumor effects in BC cells through both direct (insulin-independent) and indirect (insulin-dependent) mechanisms [24–26]. One of the well-accepted insulin-independent effects of metformin involves the activation of adenosine monophosphate activated protein kinase (AMPK), which in turn downregulates main insulin-stimulated transduction pathways, such as IRS1 and PI3K/AKT/mTOR transduction signaling [25]. Furthermore, it has been reported that IR expression status, which is frequently related to insulin levels, may represent a predictive factor of the antitumoral activity of metformin [23, 26].

Here, we provide evidence regarding novel mechanisms by which metformin may elicit anti-proliferative, anti-migratory and anti-invasive effects in a naturally immortalized BC cell line named BCAHC‐1 [25]. In particular, we show that the anti-cancer action triggered by metformin relies on its ability to inhibit the insulin/IR-mediated transduction pathway as well as the insulin-generated feedforward loop that couples CXCL12 induction by CAFs to CXCR4 expression by BCAHC‐1 cells.

## Methods

### Reagents

Insulin and Metformin were purchased from Merck Life Science (Milan, Italy). The MEK inhibitor trametinib, the insulin receptor inhibitor OSI-906 and the PI3Kα inhibitor alpelisib were obtained from MedChemExpress (DBA, Milan, Italy). The CXCR4 antagonist AMD3100 was purchased from Santa Cruz Biotechnology (DBA, Milan, Italy). All compounds were dissolved in DMSO, except insulin, metformin and AMD3100 that were solubilized in water.

### Cell cultures

BCAHC-1 cells were isolated and characterized as previously described [27], and maintained in DMEM/F-12 with phenol red, supplemented with 5% FBS and 100 μg/ml penicillin/streptomycin. Cells were grown at 37 °C in a humidified 5% CO_2_ and switched to a medium without serum and phenol red the day before treatments to be processed for experiments. CAFs were obtained as previously described [27, 28] from 10 invasive ductal breast carcinomas and pooled for the subsequent studies. Briefly, specimens were cut into 1–2 mm diameter pieces, placed in a digestion solution comprising 400 IU collagenase, 100 IU hyaluronidase, 10% serum, antibiotics, and antimycotics, and incubated overnight at 37 °C. After centrifugation at 90×*g* for 2 min, the supernatant containing fibroblasts was centrifuged at 485×*g* for 8 min; the pellet obtained was suspended in Medium 199 and Ham’s F12 mixed 1:1 (supplemented with 10% FBS and 100 μg/ml penicillin/streptomycin). CAFs were then expanded into 10-cm Petri dishes and stored as cells passaged for three population doublings within a total of 7 to tissue dissociation. Primary cell cultures of fibroblasts were characterized by immunofluorescence with human anti-vimentin (V9) and human anti-cytokeratin 14 (LL001) (Santa Cruz Biotechnology, DBA, Milan, Italy) (data not shown). FAPα antibody (H-56, Santa Cruz Biotechnology, DBA, Milan, Italy) was used to characterize activated fibroblasts (data not shown). We used CAFs passaged for up to 10 population doublings for the experiments to minimize clonal selection and culture stress, which could occur during extended tissue culture.

### Gene expression studies and PCR arrays

Total RNA was extracted, and cDNA was synthesized by reverse transcription as previously described [29]. The expression of selected genes was quantified by real-time PCR using platform Quant Studio7 Flex Real-Time PCR System (Thermo Fisher Scientific, Milan, Italy). Gene-specific primers were designed using Primer Express version 2.0 software (Applied Biosystems) and are as follows: 5′-CGAGCCCTTTGATGACTTCCT-3′ (c-Fos forward) and 5′-GGAGCGGGCTGTCTCAGA-3′ (c-Fos reverse); 5′-AGCTGTGCATCTACACCGAC-3′ (cyclin D1 forward) and 5′-GAAATCGTGCGGGGTCATTG-3′ (cyclin D1 reverse); 5′-AAGCCACCCCACTTCTCTCTAA-3′ (ACTB forward) and 5′-CACCTCCCCTGTGTGGACTT-3′ (ACTB reverse); 5′-CCTTGGAGCCAAATTTAAAACCT-3′ (CXCR4 forward) and 5′-GCTGGACCCTCTGCTCACA-3′ (CXCR4 reverse); 5′-CAGATGCCCATGCCGATTCT-3′ (CXCL12 forward) and 5′-TTCTTCAGCCGGGCTACAAT-3′ (CXCL12 reverse). Assays were performed in triplicate and the results were normalized for actin beta (ACTB) expression and then calculated as fold induction of RNA expression.

PCR arrays were performed using a TaqMan™ Human Tumor Metastasis Array (Thermo Fisher Scientific, Milan, Italy) according to the manufacturer’s instructions. The amplification reaction and the results analysis were carried out using platform Quant Studio7 Flex Real-Time PCR System (Thermo Fisher Scientific, Milan, Italy).

### Reporter gene assays

For reporter gene assays, cells (1 × 10^5^) were plated into 24-well dishes with 500 mL/well culture medium containing 5% FBS and transfected for 18 h using X-treme GENE 9 DNA Transfection Reagent, as recommended by the manufacture (Merck Life Science, Milan, Italy). A mixture containing 0.5 mg of gene reporter (Fos-luc, kindly obtained from Dr. K. Nose, Department of Microbiology, Showa University School of Pharmaceutical Sciences, Hatanodai, Shinagawa-ku, Tokyo, Japan; Cyclin D1-luc, kindly obtained from Dr. R. G. Pestell, Kimmel Cancer Center, Department of Cancer Biology and Jefferson Stem Cell and Regenerative Medicine Center, Thomas Jefferson University, Philadelphia, PA), and 5 ng of pRL-TK was then transfected, after 18 h cells were treated with insulin in the presence or absence of metformin, OSI-906, trametinib and alpelisib for additional 12 h. Luciferase activity was measured using the Dual Luciferase Kit (Promega, Milan, Italy) according to the manufacturer’s recommendations. Firefly luciferase activity was normalized to the internal transfection control provided by the Renilla luciferase activity. Normalized relative light unit values obtained from cells treated with vehicle (−) were set as onefold induction, upon which the activity induced by treatments was calculated. For gene silencing experiments, cells were plated onto 10-cm dishes and transfected by X-treme GENE 9 DNA Transfection Reagent for 24 h before treatments with a control vector and the plasmid DN/c-Fos (kindly obtained from Dr. C. Vinson, NIH, Bethesda, USA) encoding a c-Fos mutant that heterodimerizes with c-Fos dimerization partners but not allowing DNA binding.

### Western blot analysis

Cells were grown in 10-cm dishes, exposed to treatments and then lysed in 500 μl RIPA buffer with protease inhibitors (1.7 mg/ml aprotinin, 1 mg/ml leupeptin, 200 mmol/l phenylmethylsulfonyl fluoride, 200 mmol/l sodium orthovanadate and 100 mmol/l sodium fluoride). Samples were then centrifuged at 13,000 rpm for 10 min and protein concentrations were determined using BCA protein assay according to the manufacturer’s instructions (Thermo Fisher Scientific, Milan, Italy). Equal amounts of whole-protein extract were resolved on 8–10% SDS polyacrylamide gels and transferred to a nitrocellulose membrane (Merck Life Science, Milan, Italy), which were probed with primary antibodies against: phosphorylated ERK1/2 (E4), ERK2 (C-14), AKT, c-Fos (E-8), CXCR4 (4G10), Insulin Rα (N-20) and β-actin (AC-15) (Santa Cruz Biotechnology, DBA, Milan, Italy), cyclin D1 (TA801655) (OriGene Technologies, DBA, Milan, Italy), p-IR (Y1146), p-AKT (D9E) and CXCL12 (3740) (Cell Signaling Technology, Euroclone, Milan, Italy). Proteins were detected by horseradish peroxidase-linked secondary antibodies (Bio-Rad, Milan, Italy) and then revealed using the chemiluminescent substrate for western blotting Clarity Western ECL Substrate (Bio-Rad, Milan, Italy).

### Acetone precipitation of proteins

Protein precipitation from conditioned medium derived from CAFs was carried out using the precipitation method with acetone [30]. Briefly, four volumes of ice-cold acetone (Merck Life Science, Milan, Italy) were added to one volume of sample. The mixture was vortexed and incubated at − 20 °C overnight. This was followed by centrifugation at 10,000×*g* for 15 min at 4 °C. Afterwards, the supernatant was discarded, the pellet was air dried, then it was dissolved in 2× Laemmli buffer and used in the appropriate experiments. In western blot analysis, the protein loading of conditioned medium samples was checked by Ponceau red staining [0.1% Ponceau S (w/v) in 5% acetic acid] of the blotted membranes.

### Immunofluorescence microscopy

Cells were grown on a cover slip, exposed to treatments and then fixed in 4% paraformaldehyde in PBS, permeabilized with 0.2% Triton X-100, washed 3 times with PBS and incubated at 4 °C overnight with primary antibodies anti-CXCL12 (3740) (Cell Signaling Technology, Euroclone, Milan, Italy). After incubation, the slides were extensively washed with PBS, probed with Alexa Fluor conjugated secondary antibodies (Thermo Fisher Scientific, Milan, Italy) for 1 h at room temperature. Finally, cells were washed three times with PBS, incubated with DAPI (4′,6-diamidino-2-phenylindole) (1:1000) for 3 min and, after washing, immunofluorescence images were obtained using the Cytation 3 Cell Imaging Multimode reader (BioTek, AHSI, Milan Italy) and analyzed by the Gen5 software (BioTek, AHSI, Milan Italy).

### Phalloidin staining

Cells were exposed to treatments, washed twice with PBS, fixed in 4% paraformaldehyde in PBS for 10 min, washed briefly with PBS and then incubated with Phalloidin-Fluorescent Conjugate (Santa Cruz Biotechnology, DBA, Milan, Italy). The images were obtained using the Cytation 3 Cell Imaging Multimode reader (BioTek, AHSI, Milan Italy) and analyzed by the Gen5 software (BioTek, AHSI, Milan Italy).

### Cell cycle analysis

To analyze cell cycle distribution, BCAHC-1 cells (1 × 10^5^) were cultured in regular medium in 6 well plates and shifted in medium without serum. Next, BCAHC-1 cells were exposed to treatments, then pelleted, once washed with PBS and fixed in 50% methanol overnight at − 20 °C, before to stain with a solution containing 50 µg/ml propidium iodide (PI) in 1×PBS, 20 U/ml RNAse-A and 0.1% Triton (Merck Life Science, Milan, Italy). Cell phases were estimated as a percentage of a total of 10 000 events. Samples were then analyzed with CytoFLEX flow cytometry (Beckman-Coulter, Milan, Italy).

### Proliferation assay

BCAHC-1 cells (1 × 10^4^) were seeded in 24-well plates in regular growth medium, washed once they had attached, incubated in medium containing 2.5% charcoal-stripped FBS and then exposed to treatments. Treatments were renewed every day. The proliferation rate was calculated counting the cells on day 5 using the Countess Automated Cell Counter, as recommended by the manufacturer’s protocol (Thermo Fisher Scientific, Milan, Italy).

### Colony formation assay

BCAHC-1 cells were cultured in regular growth medium to 90% confluence. Cells were then trypsinized, counted and seeded (1 × 10^3^) in 6-well plates in medium containing 2.5% charcoal-stripped FBS and then exposed to treatments, as indicated. Treatments were renewed every 3 days. After 10 days, cells were washed with PBS, fixed in acetone:methanol (1:1) for 3 min at room temperature and then stained with Giemsa (Merck Life Science, Milan, Italy) for 10 min. A total of 10 pictures for each condition was detected using a digital camera and colony number was measured by ImageJ program.

### Spheroid formation assay

For spheroid generation, 100 μl/well of BCAHC-1 cell suspensions (1 × 10^4^) were placed into 2% agar coated 24-well plates in medium containing 2.5% charcoal stripped FBS. Three days after seeding, tumor spheroids (a single spheroid per well) were exposed to treatments and a 50% medium and treatment renewal was performed every 2 days. Images were obtained on day 20 using a conventional inverted microscope, thereafter cell number per spheroid was determined by trypsinizing three different spheroids, mixing the cell suspension with trypan blue and counting the number of viable cells. The total number of cells obtained was divided by the number of trypsinized spheroids.

### Conditioned medium

CAFs were placed in medium without serum and treated with vehicle (−) or 10 nM insulin for 4 h. Thereafter, CAFs were washed and fresh medium was added without serum for additional 8 h. The supernatants were then collected, centrifuged at 3500 rpm for 5 min to remove cell debris and used as conditioned medium in the appropriate experiments.

### Migration assay

Transwell 8 μm polycarbonate membranes (Costar, Merck Life Science, Milan, Italy) were used to evaluate in vitro cell migration. In 300 μl serum free medium, BCAHC-1 cells (5 × 10^4^) previously exposed to treatments were seeded in the upper chamber. Conditioned medium of CAFs was added to the bottom chambers. 8 h after seeding, cells on the upper surface of the membrane were then removed by wiping with Q-tip, and migrated cells were fixed with 100% methanol, stained with Giemsa (Merck Life Science, Milan, Italy), photographed using a digital camera and counted using the WCIF ImageJ software.

### Matrigel evasion assay

The Matrigel drop assay was performed as previously described [31, 32] and modified by suspending 3 × 10^4^/drop of BCAHC-1 cells, previously exposed to treatments, in 15 μl of complete medium and gently mixed with 15 μl of Corning® Matrigel® Growth Factor Reduced (GFR) Basement Membrane Matrix (Biogenerica, Catania, Italy). The cell/matrigel suspension was layered onto the surface of 12-well plate to form a well-defined drop and placed at 37 °C to solidify. 2 ml of conditioned media collected from CAFs previously treated with vehicle (−) or 10 nM insulin were placed over the drop. Cells were observed at specified time points and drops were photographed using the ImageFocus Plus V2 software of the inverted Oxion Inverso microscope (Euromex Microscopen bv, The Netherlands). The number of cells migrated out of the drop was measured.

### TCGA database and data collection

Bioinformatics studies were performed using the Invasive Breast Cancer Cohort data of the publicly available dataset The Cancer Genome Atlas (TCGA). The patient clinical information, gene expression data (RNA Seq V2 RSEM) and the pathway activity (z-scores) were retrieved from UCSC Xena (https://xenabrowser.net/) on the 7th March 2022. Samples (n. 1247) were filtered for missing values.

### Survival analysis

The survival analyses were performed using the IR-mediated signaling pathway activity and the IR and CXCR4 gene expression data of the TCGA patients along with the overall survival (OS), and disease-free interval (DFI) information. The survivALL package was employed to examine Cox proportional hazards for all possible points of separation (low–high cut-points). The cut-point with the lowest p-value was selected [33], therefore dividing the patients with high (n = 488) and low (n = 87) IR-mediated signaling pathway activity, high (n = 166) and low (n = 90) IR expression or high (n = 145) and low (n = 107) CXCR4 expression. The Kaplan Meier survival curves were generated using the R survival and survminer packages.

### Statistical analysis

The statistical analysis was performed using ANOVA followed by NewmanKeuls’ test to determine differences in means. Densitometric analysis was performed using the freeware software ImageJ that allowed to quantify the band intensity of the protein of interest with respect to the band intensity of the loading control. All bioinformatics analyses were carried out using R Studio. Box plots were performed with the tidyverse package and the related statistical analysis was carried out by using the t-test. Heatmaps were drawn on the log_2_ fold changes of gene expression using the pheatmap package. p‐values < 0.05 were considered statistically significant.

## Results

### Metformin prevents the growth effects triggered by insulin/IR signaling in BCAHC-1 cells

**Fig. 1:**
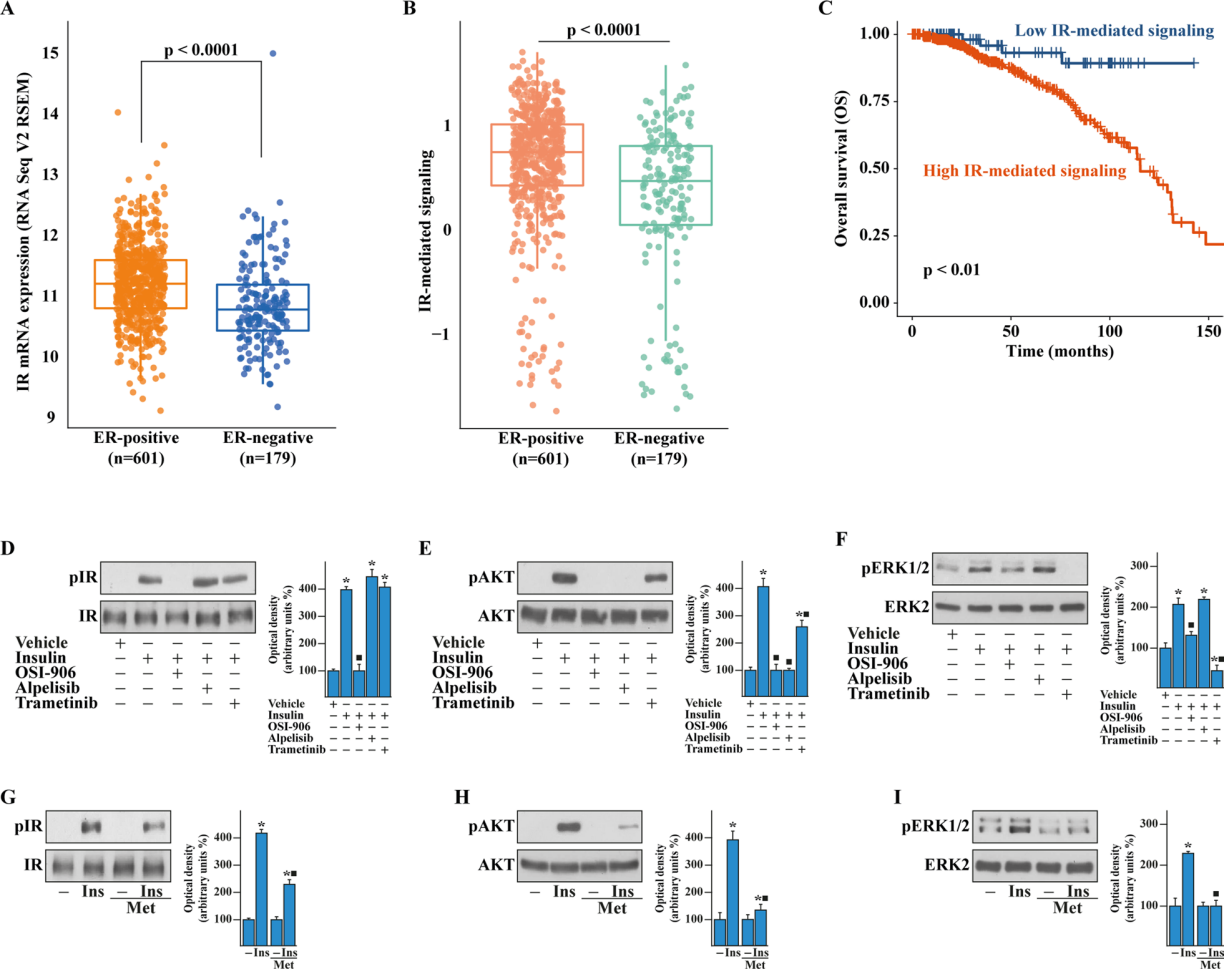
Assessment of IR signaling. Box plots depicting the expression of insulin receptor (IR) (**A**) and IR-mediated signaling pathway (**B**) in ER-positive and ER-negative breast cancer (BC) patients of the TCGA cohort. **C** Kaplan–Meier curve showing the correlation of IR-mediated signaling pathway with overall survival (OS) of ER-positive BC patients of the TCGA dataset. p-values are reported in each panel. Protein levels of phosphorylated IR (pIR) (**D**), AKT (pAKT) (**E**) and ERK (pERK1/2) (**F**) in BCAHC-1 cells exposed for 30 min to vehicle or 10 nM insulin in the presence or absence of 1 µM IR inhibitor OSI-906, 1 µM PI3K inhibitor alpelisib and 100 nM MEK inhibitor trametinib, as indicated. Protein levels of pIR (**G**), pAKT (**H**) and pERK1/2 (**I**) in BCAHC-1 cells treated with vehicle (−) or 10 nM insulin (Ins) for 30 min alone or in combination with 2 mM metformin (Met), which was added to culture medium 18 h before the treatment with vehicle or insulin. Side panels show densitometric analysis of the blots normalized to IR, AKT and ERK2 that served as loading control, as indicated. Results shown are representative of at least three independent experiments. (*) indicates significant differences with respect to vehicle sample (p < 0.05); (black square) indicates significant differences with respect to Ins treated sample (p < 0.05)

**Fig. 2:**
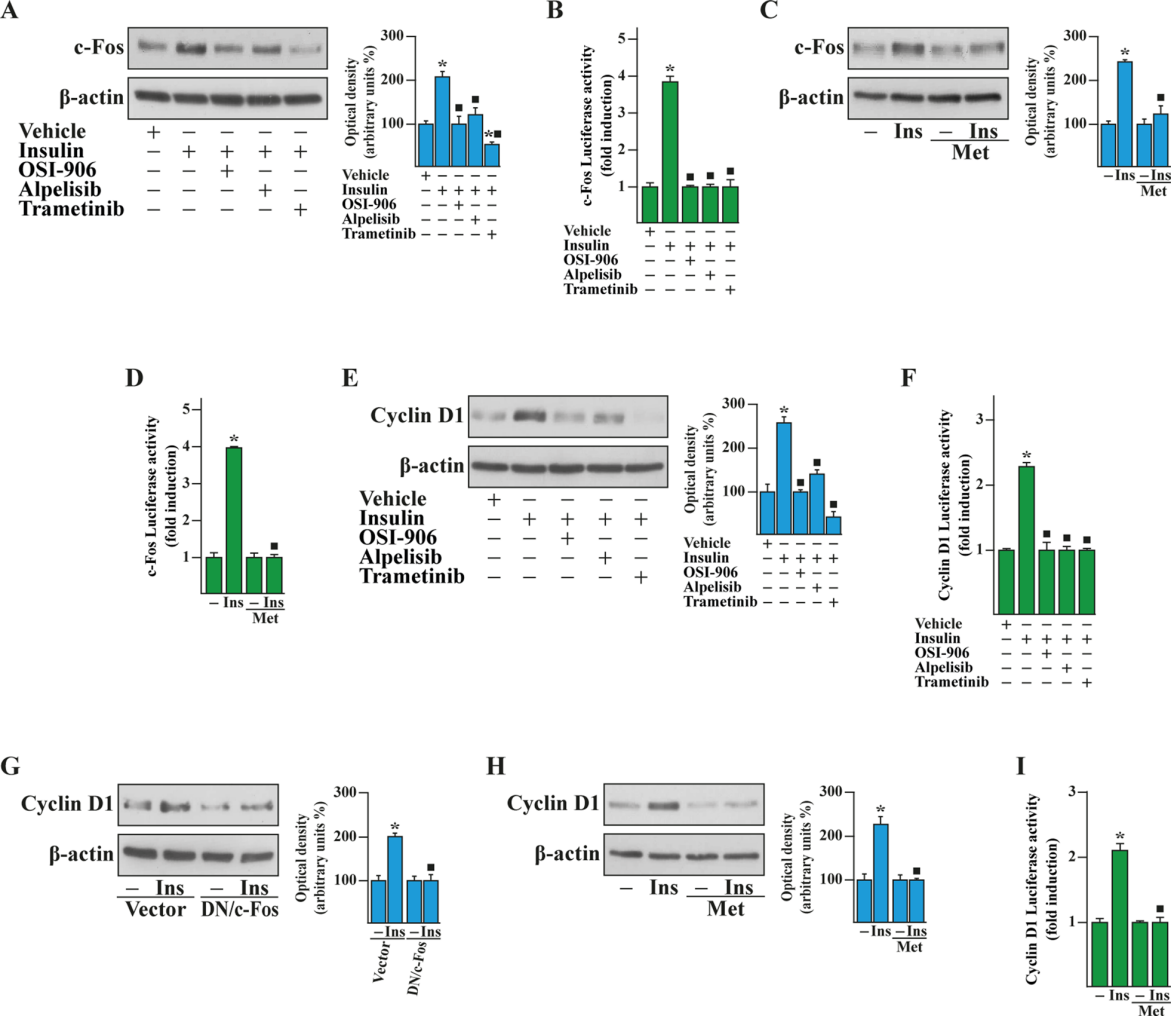
Metformin abrogates c-Fos and Cyclin D1 expression induced by insulin in BCAHC-1 cells. **A** Immunoblots of c-Fos from BCAHC-1 cells treated for 6 h with vehicle (−) or 10 nM insulin (Ins) in the presence or absence of 1 µM IR inhibitor OSI-906, 1 µM PI3K inhibitor alpelisib or 100 nM MEK inhibitor trametinib. **B** BCAHC-1 cells were transiently transfected for 18 h with a c-Fos promoter construct, then were treated for 12 h with vehicle or 10 nM Ins in the presence or absence of 1 µM IR inhibitor OSI-906, 1 µM PI3K inhibitor alpelisib or 100 nM MEK inhibitor trametinib. **C** Protein levels of c-Fos in BCAHC-1 cells exposed for 6 h to vehicle or 10 nM Ins alone or in combination with 2 mM metfomin (Met), which was added to culture medium 18 h before the treatment with vehicle or insulin. **D** BCAHC-1 cells were transiently transfected for 18 h with c-Fos and then cells were treated for 12 h with vehicle or 10 nM Ins alone or in combination with 2 mM Met, which was added to culture medium 18 h before the treatment with vehicle or insulin. **E** Immunoblots of Cyclin D1 from BCAHC-1 cells treated for 6 h with vehicle or 10 nM Ins in the presence or absence of 1 µM IR inhibitor OSI-906, 1 µM PI3K inhibitor alpelisib or 100 nM MEK inhibitor trametinib. **F** BCAHC-1 cells were transiently transfected for 18 h with a Cyclin D1 promoter construct, then cells were treated for 12 h with vehicle or 10 nM Ins in the presence or absence of 1 µM IR inhibitor OSI-906, 1 µM PI3K inhibitor alpelisib or 100 nM MEK inhibitor trametinib. **G** Immunoblots of Cyclin D1 from BCAHC-1 cells transfected for 24 h with a vector or a dominant-negative c-Fos construct (DN/c-Fos) and then exposed for 6 h to vehicle or 10 nM Ins. **H** Protein levels of Cyclin D1 in BCAHC-1 cells exposed for 6 h to vehicle or 10 nM Ins alone or in combination with 2 mM Met, which was added to culture medium 18 h before the treatment with vehicle or insulin. Side panels show densitometric analysis of the blots normalized to β-actin. Results shown are representative of at least three independent experiments. **I** BCAHC-1 cells were transiently transfected for 18 h with a Cyclin D1 promoter construct, then cells were treated for 12 h with vehicle or 10 nM Ins alone or in combination with 2 mM Met, which was added to culture medium 18 h before the treatment with vehicle or insulin. The luciferase activities were normalized to the internal transfection control, and values of cells receiving vehicle were set as onefold induction on which the activity induced by treatments was calculated. Columns represent the mean ± SD of three independent experiments performed in triplicate. (*) indicates significant differences with respect to vehicle sample (p < 0.05); (black square) indicates significant differences with respect to Ins treated sample (p < 0.05)

**Fig. 3:**
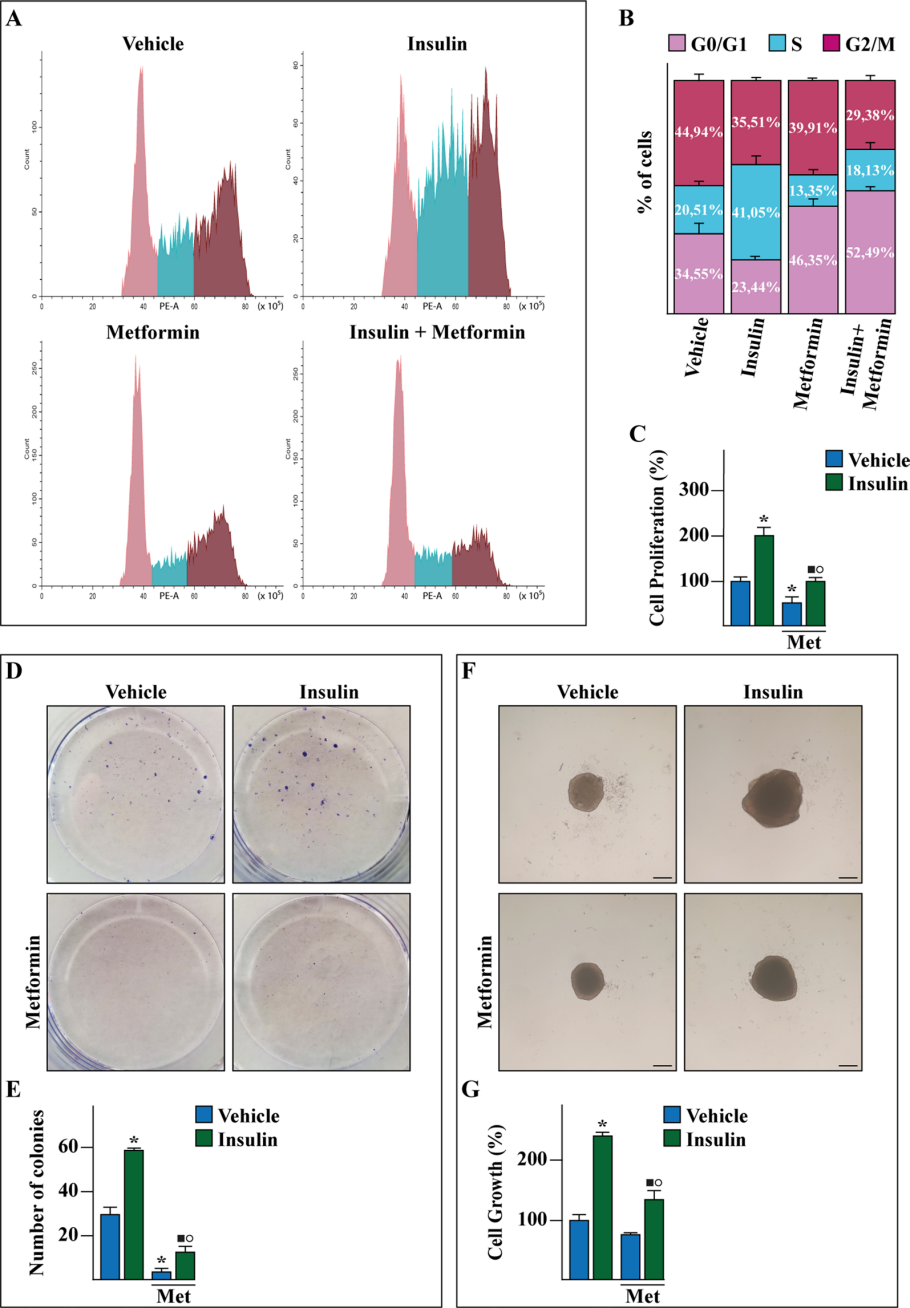
Growth-inhibitory effects of metformin in BCAHC-1 cells. **A** Cell cycle analysis performed by flow cytometry in BCAHC-1 cells treated with vehicle or 10 nM insulin for 6 h alone or in combination with 2 mM metformin, which was added to culture medium 18 h before the treatment with vehicle or insulin. **B** Percentage of BCAHC-1 cells in G0/G1, S and G2/M phases of cell cycle. The values represent the mean ± SD of three independent experiments. **C** Proliferation of BCAHC-1 cells after 5 days treatment with vehicle or 10 nM insulin alone or in combination with 2 mM metformin (Met), which was added to culture medium 18 h before the treatment with vehicle or insulin. Values of cells treated with vehicle were set as 100% upon which proliferation induced by treatments was determined. **D** Colony formation assay in BCAHC-1 cells exposed to vehicle or 10 nM insulin alone or in combination with 2 mM metformin, which was added to culture medium 18 h before the treatment with vehicle or insulin. The plates were stained with Giemsa and colonies were counted following 10 days of incubation (**E)**. **F** Representative pictures of spheroids (a single spheroid/well) grown on agar-coated plates upon 20 days treatment with vehicle or 10 nM insulin alone or in combination with 2 mM metformin. Scale bar: 100 μm. **G** Quantification of BCAHC-1 spheroid growth. The number of cells treated with vehicle was set as 100% upon which the number of cells upon treatments was determined. Each data point is the mean ± SD of three independent experiments performed in triplicate. (*) indicates significant differences with respect to vehicle sample (p < 0.05); (black square) indicates significant differences with respect to Insulin treated sample (p < 0.05); (white circle) indicates significant differences with respect to Metformin treated sample (p < 0.05)

Previous studies have shown that a crosstalk between estrogen and insulin transduction pathways promotes the progression of BC [27, 34, 35]. Therefore, we began our study analyzing the expression and activity of IR in the TCGA cohort of ER-positive and negative BC patients. First, we ascertained that the IR mRNA expression as well as the IR-mediated signaling pathway are significantly higher in ER-positive than ER-negative BCs (Fig. 1A, B). In addition, stratifying the BC patients on the basis of the PAM50 gene signature, both IR expression and signaling were found higher in the BC luminal A and luminal B patients respect to the other BC subtypes (Additional file 1: Fig. S1A, B). In accordance with these data, was found enriched and associated with a worse overall survival (OS) (Fig. 1C) in ER-positive respect to ER-negative BC patients. Pre‑clinical and currently ongoing clinical trials indicate that the insulin-sensitizer metformin elicits anti-tumor effects in ER-positive BCs and is associated with a lower risk of developing BC in patients with T2D respect to control patients [11, 20, 36–40]. Hence, we aimed to ascertain whether metformin may inhibit insulin-dependent signaling pathways like phosphoinositide 3-kinase (PI3K)/AKT and mitogen-activated protein kinase (MAPK). To this end, we used as a model system a BC cell line namely BCAHC-1, which is characterized by the peculiar expression of IR and the 46 kDa ERα splice variant (ERα46) [27, 41, 42]. First, the insulin-induced IR phosphorylation was prevented using the IR inhibitor OSI-906, but not in the presence of the PI3K inhibitor alpelisib and the MEK inhibitor trametinib (Fig. 1D). The activation of AKT upon insulin exposure was totally abrogated by OSI-906 and alpelisib, but only partially dampened by trametinib (Fig. 1E) as previously reported [43], whereas the insulin-promoted activation of the ERK1/2 pathway was inhibited by OSI-906 and trametinib but not using alpelisib (Fig. 1F). Next, we assessed that metformin reduces the IR phosphorylation prompted by insulin, without any change in total IR protein expression (Fig. 1G). Moreover, we determined that metformin prevents the activation of both AKT and ERK1/2 upon insulin exposure in BCAHC-1 cells (Fig. 1H, I). On the basis of these findings and in line with previous studies showing that PI3K/AKT and ERK1/2 transduction pathways mediate the expression of insulin target genes, such as c‐Fos and Cyclin D1 [27, 44–46], we found that insulin induces c-Fos expression in BCAHC-1 cells at both mRNA (data not shown) and protein levels (Fig. 2A–C). Worthy, OSI-906, alpelisib, trametinib and metformin abrogated either the protein increase (Fig. 2A–C) or the activation of a promoter construct of c-Fos transiently transfected in BCAHC-1 cells (Fig. 2B–D). Similarly, we established that OSI-906, alpelisib and trametinib prevent the Cyclin D1 protein increase and the transactivation of a Cyclin D1 promoter construct upon insulin exposure (Fig. 2E, F). Reminiscing our previous findings showing that c-Fos is involved in the regulation of Cyclin D1 [27], we demonstrated that the protein induction of Cyclin D1 by insulin is prevented transfecting BCAHC-1 cells with a DN/c-Fos expression vector (Fig. 2G). Furthermore, we showed that metformin abolishes the Cyclin D1 protein increase (Fig. 2H) and the activation of a Cyclin D1 promoter construct (Fig. 2I) triggered by insulin. Taken together, these findings indicate that in BCAHC-1 cells metformin inhibits the insulin-induced IR/PI3K/MAPK/c-Fos transduction pathway, thus preventing the up-regulation of Cyclin D1. In line with the acknowledged role of Cyclin D1 as a regulator of G1/S transition in the cell cycle [47], we found that metformin leads to the arrest within the G0/G1 phase of the cell cycle in BCAHC-1 cells (Fig. 3A, B). Interestingly, the results of flow cytometric analysis of propidium iodide (PI)-staining showed that insulin induces S-phase entry in BCAHC-1 cells, however this effect is no longer evident in the presence of metformin (Fig. 3A, B) and using OSI-906, alpelisib or trametinib (data not shown). In accordance with these observations, the proliferation and colony-forming ability of BCAHC-1 cells upon insulin treatment were abolished by metformin (Fig. 3C–E) as well as in the presence of OSI-906, alpelisib, trametinib (Additional file 2: Fig. S2A–C). In addition, the spheroid expansion of BCAHC-1 cells prompted by insulin was inhibited by metformin (Fig. 3F, G) as well as OSI-906, alpelisib and trametinib (Additional file 2: Fig. S2D, E). Overall, these data indicate that metformin impairs the growth responses induced by insulin in BCAHC-1 cells.

### Metformin inhibits the up-regulation of the pro-tumorigenic chemokine receptor CXCR4 triggered by insulin in BCAHC-1 cells

**Fig. 4:**
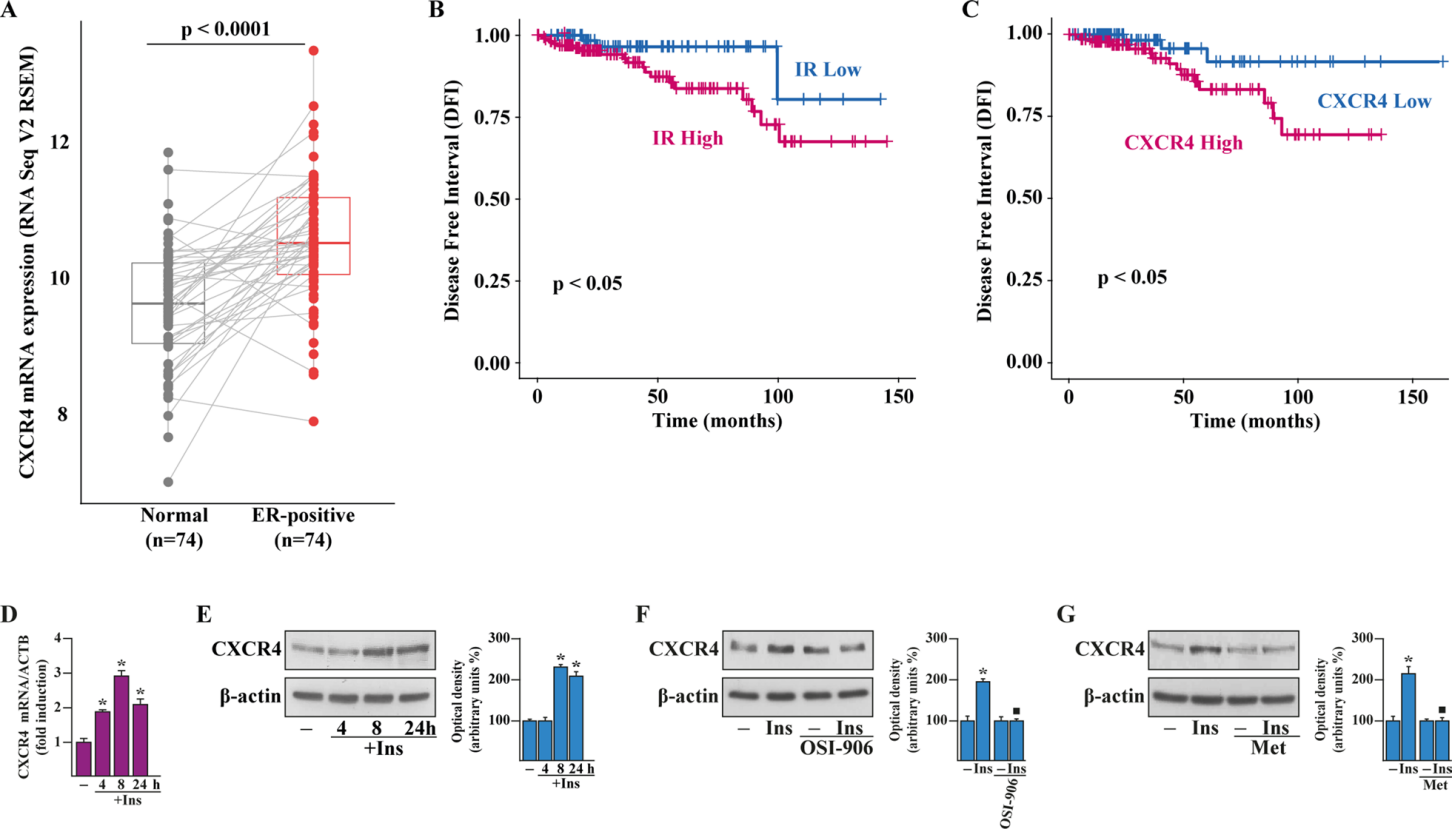
CXCR4 is associated with poor outcome in ER-positive BC and stimulated by insulin. **A** Pairwise comparison of CXCR4 expression in ER-positive breast cancer (BC) and the adjacent normal tissues of the TCGA dataset. **B** Kaplan–Meier plot showing the correlation between insulin receptor (IR) expression and disease-free interval (DFI) in ER-positive BC patients exhibiting CXCR4 levels above the median value. **C** Kaplan–Meier plot showing the correlation of CXCR4 expression with DFI in ER-positive BC patients exhibiting IR levels above the median value. mRNA (**D**) and protein (**E**) levels of CXCR4 evaluated respectively by real-time PCR and immunoblotting in BCAHC-1 cells exposed to vehicle (−) or 10 nM insulin (Ins), as indicated. In RNA experiments, values are normalized to the actin beta (ACTB) expression and presented as fold changes of mRNA expression upon treatments relative to vehicle. **F** Protein levels of CXCR4 in BCAHC-1 cells exposed for 8 h to vehicle or 10 nM Ins alone or in combination with 1 µM IR inhibitor OSI-906. **G** Immunoblot of CXCR4 from BCAHC-1 cells treated for 8 h with vehicle or 10 nM Ins alone or in combination with 2 mM metformin (Met), which was added to culture medium 18 h before the treatment with vehicle or insulin. Side panels show densitometric analysis of the blots normalized to the loading control. Results shown are representative of at least three independent experiments. (*) indicates significant differences with respect to vehicle sample (p < 0.05); (black square) indicates significant differences with respect to Insulin treated sample (p < 0.05)

Considering that a negative correlation between metformin and the metastatic spread of BC has been reported [48–50], we then assessed whether metformin may abolish the capability of insulin to induce a metastatic gene signature in BCAHC-1 cells. In this vein, we performed a TaqMan Gene Expression Assay consisting of a Human Tumor Metastasis Array in BCAHC-1 cells exposed to insulin in the presence or absence of metformin. Among the genes that showed at least a 0.25 log2 fold change upon insulin treatment respect to vehicle and the reduction of this increase in the presence of metformin, we focused on the C-X-C Motif Chemokine Receptor 4 (CXCR4) due to its acknowledged role as prognostic marker and main driver of BC metastasis [51–53]. Evaluating the expression of CXCR4 in ER-positive BC samples of the TCGA dataset, we assessed by pairwise comparison that CXCR4 levels are significantly higher in ER-positive BCs respect to matched normal tissues (Fig. 4A). Thereafter, we ascertained whether the mRNA expression of both CXCR4 and IR would be predictive of the outcome of ER-positive BC patients. In this regard, survival analyses revealed that high IR levels are associated with a short disease-free interval (DFI) in BC patients showing increased CXCR4 expression (Fig. 4B). Similarly, a correlation between high CXCR4 levels and poor prognosis characterizes BCs displaying enhanced IR levels (Fig. 4C). Considering that insulin modulates pro-metastatic mediators involved in the regulation of CXCR4 in BC cells [54, 55], we ascertained that insulin increases both mRNA (Fig. 4D) and protein levels (Fig. 4E) of CXCR4 in BCAHC-1 cells. Next, immunoblot experiments showed that either the IR inhibitor OSI-906 (Fig. 4F) or metformin (Fig. 4G) prevent the up-regulation of CXCR4 triggered by insulin, suggesting that metformin prevents the expression of CXCR4 mediated by insulin/IR axis in BCAHC-1 cells.

### Insulin induces the secretion of the CXCR4 ligand CXCL12 in cancer-associated fibroblasts (CAFs)

**Fig. 5:**
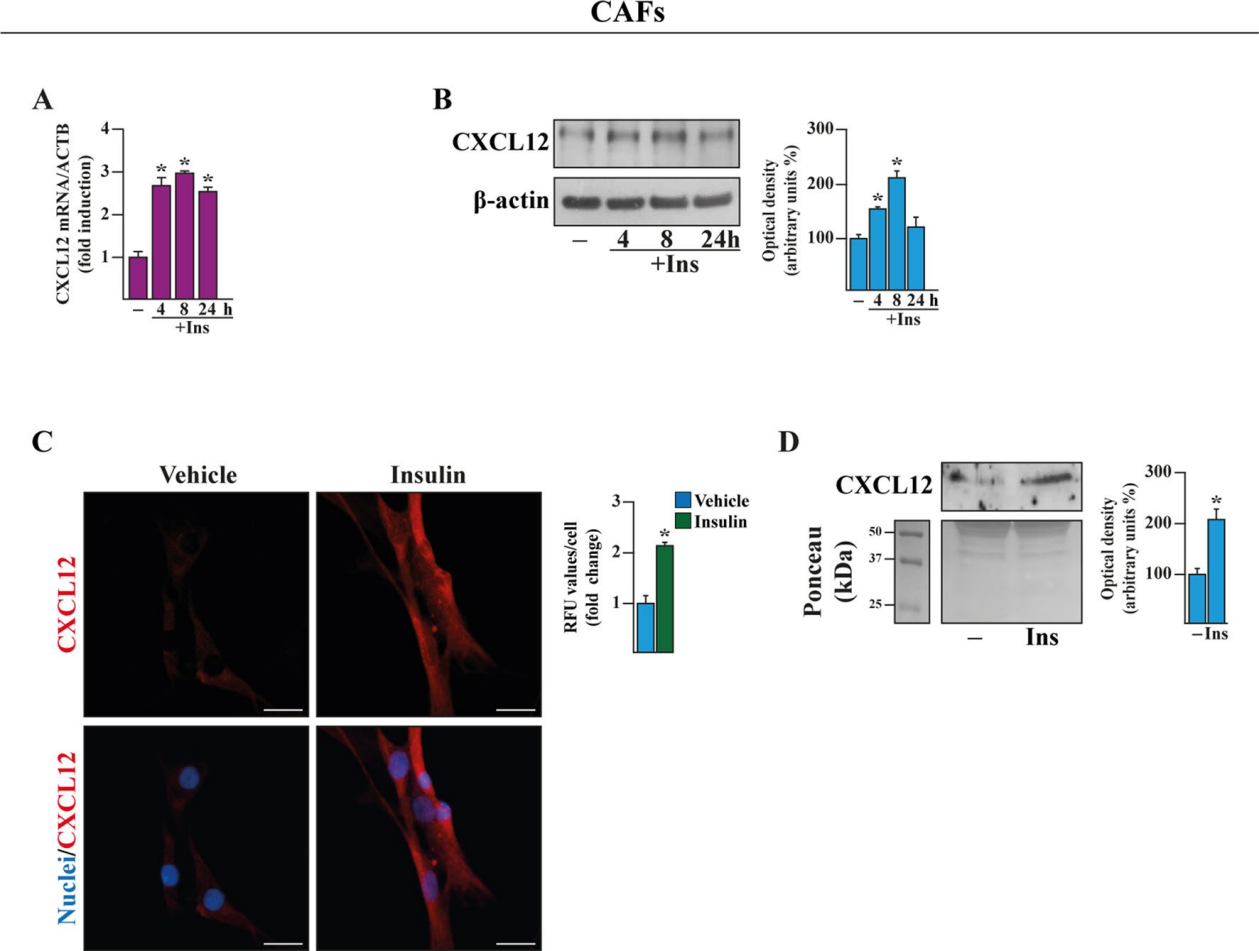
Insulin triggers CXCL12 expression in cancer-associated fibroblasts (CAFs). mRNA (**A**) and protein (**B**) expression of CXCL12 evaluated respectively by real-time PCR and immunoblotting in CAFs exposed to vehicle (−) or 10 nM insulin (Ins), as indicated. In RNA experiments, values were normalized to actin beta (ACTB) expression and shown as fold changes of mRNA expression upon treatments respect to vehicle. **C** CXCL12 expression evaluated by immunofluorescence assays in CAFs treated with vehicle or 10 nM insulin for 8 h. Nuclei were stained by DAPI (blue signal). Fluorescence intensities were quantified in 10 random fields for each condition and results are expressed as fold change of relative fluorescence units (RFU) over cells treated with vehicle (set as one-fold change). Scale bar: 25 μm. **D** Assessment of CXCL12 protein levels in conditioned medium (CM) collected from CAFs treated for 8 h with vehicle (−) and 10 nM Ins. Ponceau red staining was used as a loading control for CM. Side panels show densitometric analysis of the blots normalized to the respective loading control. Results shown are representative of at least three independent experiments. (*) indicates significant differences with respect to vehicle (−) sample (p < 0.05)

A crosstalk between tumor cells and cancer‐associated fibroblasts (CAFs) may fuel the growth and metastasis of BC [56–59]. In particular, the CAF-secreted CXCR4 ligand, namely CXCL12 (also known as SDF-1), is an important factor linking stromal and BC cells toward aggressive malignant features [59–64]. On the basis of the aforementioned findings and considering that insulin has been implicated in the release of pro-tumorigenic and inflammatory mediators by stromal cells [65, 66], we ascertained whether insulin may promote the expression and secretion of CXCL12 in breast CAFs. By real-time PCR, immunoblotting and immunofluorescence experiments we demonstrated that the expression of CXCL12 increases at both mRNA (Fig. 5A) and protein levels (Fig. 5B, C) in CAFs exposed to insulin. Moreover, CXCL12 was found up-regulated in conditioned medium (CM) of CAFs treated with insulin (Fig. 5D), as ascertained by acetone precipitation assays. Altogether, these data suggest that insulin induces the increase of the CXCR4 ligand CXCL12 and stimulates its release by CAFs.

### Metformin suppresses the insulin-driven feedforward loop linking CXCR4 induction in BCAHC-1 cells to CXCL12 expression and secretion by CAFs

**Fig. 6:**
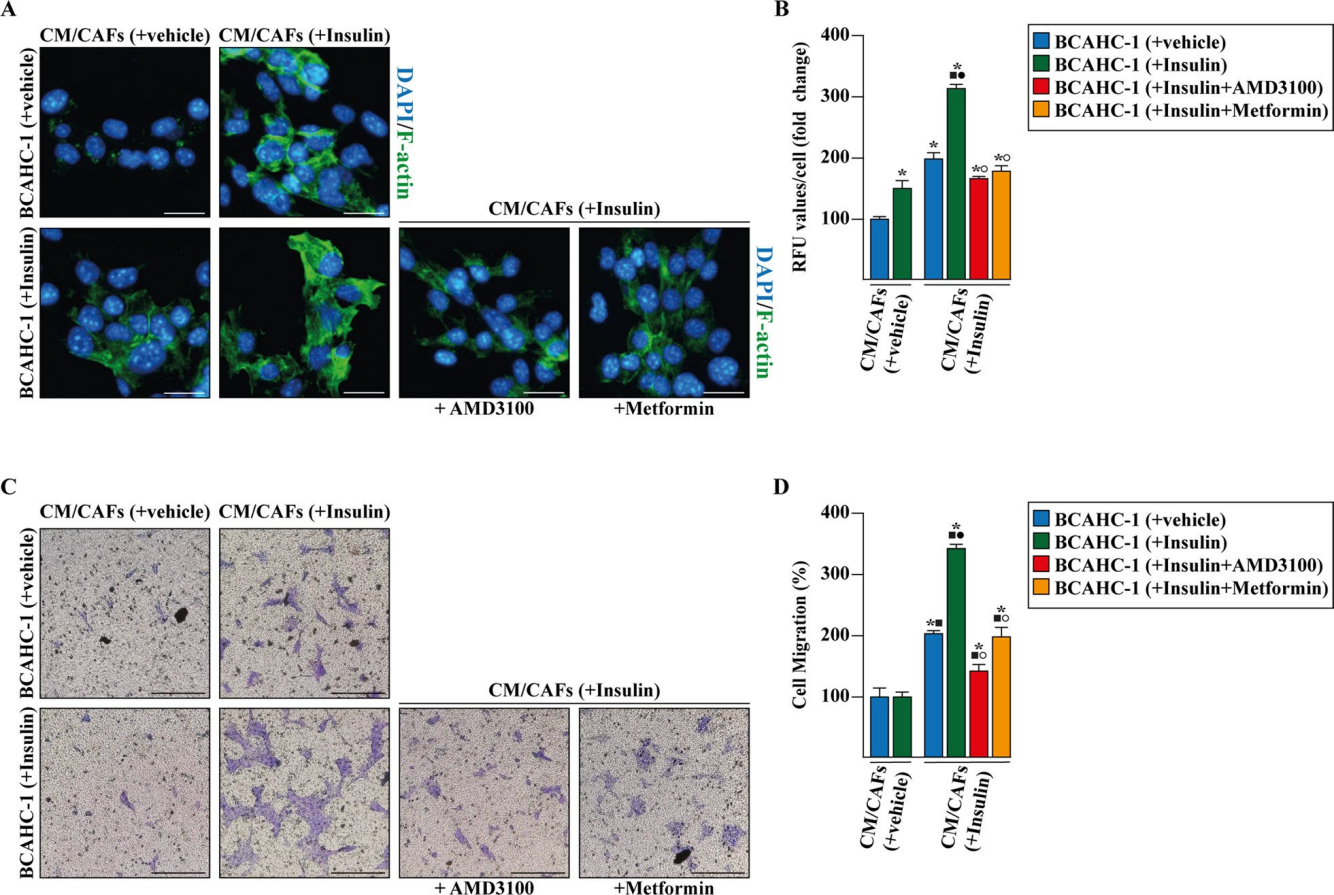
Insulin-stimulated CXCL12/CXCR4 axis promotes the cytoskeleton reorganization and migration in BCAHC-1 cells. **A** FITC-phalloidin staining of BCAHC-1 cells treated for 8 h with vehicle or 10 nM insulin alone or in combination with 10 μM CXCR4 antagonist AMD3100 or 2 mM metformin that was added to culture medium 18 h before the treatments. Cells were then cultured for additional 8 h to conditioned medium (CM) collected from CAFs, which were previously treated for 8 h with vehicle or 10 nM insulin, as indicated. Cells were stained with FITC-phalloidin to detect F-actin stress fibers (green) and with DAPI to detect nuclei (blue). **B** Fluorescence intensities of the number of stress fibers/cell was quantified based F-actin staining in 10 random fields for each treatment; results are shown as fold change of relative fluorescence units (RFU). Scale bar: 25 μm. Values represent the mean ± SD of three independent experiments performed in triplicate. (*) indicates p < 0.05 for cells exposed to treatments versus vehicle. **C** Transwell migration assay in BCAHC-1 cells treated for 8 h with vehicle or 10 nM insulin alone or in combination with 10 μM CXCR4 antagonist AMD3100 or 2 mM metformin that was added to culture medium 18 h before the treatments. Cells were then cultured for additional 8 h to conditioned medium (CM) collected from CAFs, which were previously treated for 8 h with vehicle or 10 nM insulin, as indicated. **D** Evaluation of cell migration in 10 random fields in each of three independent experiments performed in triplicate. Scale bar: 200 μm. (*) indicates significant differences with respect to BCAHC-1 (+vehicle) exposed to CM/CAFs (+vehicle) sample (p < 0.05); (black square) indicates significant differences with respect to BCAHC-1 (+Insulin) exposed to CM/CAFs (+vehicle) sample (p < 0.05); (black circle) indicates significant differences with respect to BCAHC-1 (+vehicle) exposed to CM/CAFs (+Insulin) sample (p < 0.05); (white circle) indicates significant differences with respect to BCAHC-1 (+Insulin) exposed to CM/CAFs (+Insulin) sample (p < 0.05)

**Fig. 7:**
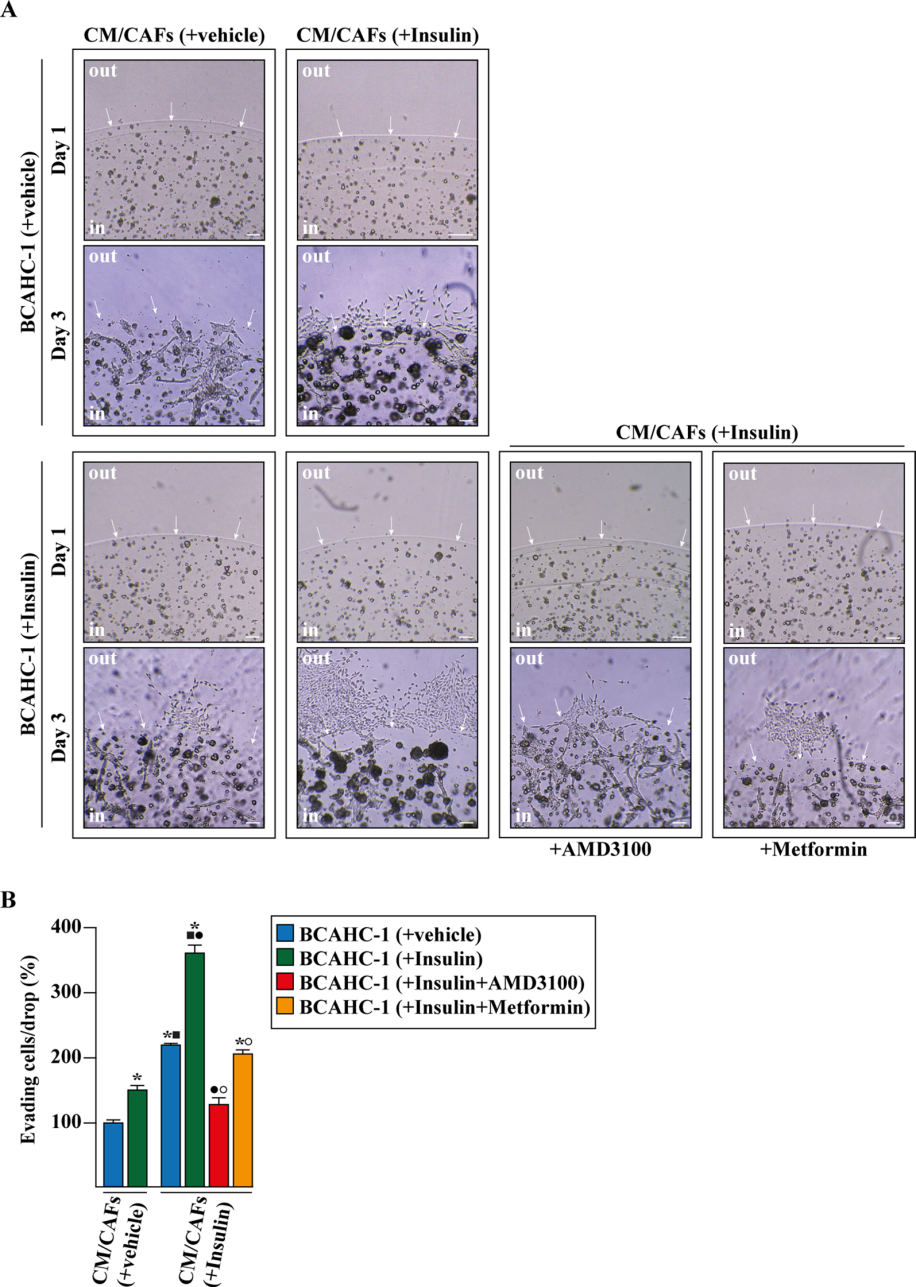
Insulin-stimulated CXCL12/CXCR4 signaling triggers matrigel evasion of BCAHC-1 cells. **A** Representative pictures from the matrigel drops evasion assay in BCAHC-1 cells treated for 8 h with vehicle or 10 nM insulin alone or in combination with 10 μM CXCR4 antagonist AMD3100 or 2 mM metformin that was added to culture medium 18 h before the treatments. Cells were then cultured for additional 3 days to conditioned medium (CM) collected from CAFs, which were previously treated for 8 h with vehicle or 10 nM insulin, as indicated. Arrows point to the matrigel drop border. **B** Percentage of cells around the drop upon 3 days treatment from three independent experiments performed in triplicate. Scale bar: 500 μm. (*) indicates significant differences with respect to BCAHC-1 (+vehicle) exposed to CM/CAFs (+vehicle) sample (p < 0.05); (black square) indicates significant differences with respect to BCAHC-1 (+Insulin) exposed to CM/CAFs (+vehicle) sample (p < 0.05); (black circle) indicates significant differences with respect to BCAHC-1 (+vehicle) exposed to CM/CAFs (+Insulin) sample (p < 0.05); (white circle) indicates significant differences with respect to BCAHC-1 (+Insulin) exposed to CM/CAFs (+Insulin) sample (p < 0.05)

Considering the pivotal role played by the CXCL12/CXCR4 axis in BC development and metastasis [51, 64, 67, 68], we evaluated whether insulin may engage the CXCL12/CXCR4 signaling, leading to aggressive features in BCAHC-1 cells. Hence, in order to visualize the F-actin pattern CM from CAFs exposed to vehicle and insulin was collected and used as culture medium in BCAHC-1 cells, which were previously treated with vehicle and insulin. These experimental conditions allowed us to observe a marked increase in F-actin assembly in BCAHC-1 cells treated with insulin and cultured with CM from insulin-stimulated CAFs (Fig. 6A, B). These effects were prevented using the specific CXCR4 antagonist AMD3100 and metformin (Fig. 6A, B). The aforementioned findings were corroborated by transwell migration assays performed in BCAHC-1 cells that were previously treated with insulin and cultured as described above. Of note, the migration of BCAHC-1 cells prompted by insulin was further potentiated culturing cells with CM obtained from insulin-treated CAFs (Fig. 6C, D), however this response was no longer evident treating BCAHC-1 cells with AMD3100 and metformin (Fig. 6C, D). Similarly, three-dimensional motility assays showed that AMD3100 and metformin prevent the outflow from the matrigel drop of insulin-treated BCAHC-1 cells cultured with CM from insulin-stimulated CAFs (Fig. 7A, B). Taken together, these results suggest that the insulin-activated CXCL12/CXCR4 axis sustains a paracrine feedforward loop coupling cancer and stromal cells, thus fostering a motile phenotype in BCAHC-1 cells.

## Discussion

Insulin-resistance and related metabolic disorders, such as obesity, T2D and metabolic syndrome, contribute to cancer development and progression dysregulating many oncogenic pathways mediated by hormones, growth factors and cytokines [69, 70]. For instance, an early indicator of metabolic dysfunction as hyperinsulinemia, has been considered an independent risk and prognostic factor for BC [71–73]. In this regard, insulin has been shown to facilitate BC progression through multiple signaling pathways that promote mitogenic and metastatic responses [13, 74, 75]. An additional insight toward a better understanding of insulin action in BC derives from the observation that the IR isoform A (IR-A) is frequently overexpressed in BC patients [17]. Contrary to the isoform B (IR-B) that is predominantly expressed in adult tissues and mediates metabolic effects of insulin, IR-A is involved in fetal development and is re-expressed in malignant tissues, where it triggers the oncogenic responses to insulin [17, 18, 76]. In addition, it should be mentioned that the dysregulation of the IR-A/IR-B ratio may be involved in pro-tumorigenic effects elicited by insulin [17, 77, 78]. In this scenario, we have recently isolated a naturally immortalized BC cell line named BCAHC‐1, which is distinguished by a peculiar receptor expression profile that may allow to better dissect the mechanisms underlying the stimulatory effects of insulin/IR-A axis in BC [27]. Considering that insulin and IGFs are able to bind to and activate each other’s receptors and share common downstream signaling pathways [78, 79], BCAHC‐1 cells may represent a valuable model system for a comprehensive evaluation of insulin/IR signaling in BC.

Metformin (1,1-dimethylbiguanide hydrochloride) is the most commonly prescribed drug for T2D treatment worldwide, displaying a remarkable balance between efficacy and safety profile [80]. The effectiveness of metformin in improving the sensitivity to insulin, and therefore in lowering insulin circulating levels, may provide a rationale to investigate the potential benefits of metformin in cancer patients affected by metabolic disorders [81]. As it concerns BC, several preclinical findings have demonstrated that metformin can interfere with cell proliferation and induce cell cycle arrest and apoptosis [25, 82]. Moreover, metformin may modify the expression of main pro-tumorigenic genes and exert a synergic action with chemotherapeutics in BC cells [83–87]. Therefore, metformin has been suggested as a potential antitumoral agent in BC also considering the results of epidemiological studies in both diabetic and non-diabetic women [20, 26, 88–90]. Cumulatively, these data recently paved the way for numerous trials aimed at investigating whether patients with diverse BC subtypes may take advantage from the use of metformin either alone or in combination with chemotherapeutics. In this regard, a recent meta-analysis revealed an increased objective response rate (ORR) in metformin-treated patients with inoperable BCs [91]. Moreover, data from a prospective study evidenced that the use of metformin is associated with a decreased risk of progression in ER-positive BC associated with T2D, whereas this beneficial effect was absent in ER-negative BC associated with T2D [40]. These investigations may suggest that ER is involved in the reduced risk of BC by metformin, likely through the inhibition of either ER expression or ER-mediated transcriptional activity [92]. Accordingly, we have previously demonstrated that a bi-directional crosstalk between ERα46 and IR may occur in BCAHC‐1 cells upon estrogen and insulin stimulation toward growth and pulmonary metastases [27], in line with the cooperation between ERα and IR transduction pathways assessed in BC cells [34, 35, 93].

In this study, we provide novel insights on the ability of metformin to prevent BC growth and motility stimulated by insulin/IR signaling. First, an integrated bioinformatics analysis allowed us to show that the expression and activation of IR are both associated with a worse outcome in ER-positive respect to ER-negative BC patients. Next, we focused on the characterization of the molecular mechanisms by which metformin might inhibit the stimulatory responses triggered by the insulin/IR axis in an IGF-deficient model system as BCAHC‐1 cells. In line with previous evidence demonstrating that metformin lowers IR phosphorylation [94], we observed that metformin inhibits the insulin-dependent activation of IR in BCAHC‐1 cells, without changes in IR expression. Previous studies have also reported a reduction or even an increase of IR protein levels upon metformin treatment, suggesting a differential regulation of IR expression depending on the cell context [95–97]. We next ascertained that in BCAHC‐1 cells metformin prevents the activation of two main insulin-stimulated signaling pathways, namely PI3K/AKT and MAPK [41, 42], leading to the downregulation of insulin target genes like c-Fos and Cyclin D1 [27]. In accordance with these data, metformin was shown to down-regulate the expression of transcription factors as well as cell-cycle regulators [98, 99]. Nicely recapitulating these observations, we found that metformin inhibits the proliferative responses triggered by insulin in BCAHC-1 cells, including cell cycle progression, spheroids expansion and colony formation. Notably, metformin increased the proportion of BCAHC-1 cells in G0/G1 phase, reduced cell growth and inhibited the rate of colony formation. Reminiscing previous studies revealing that metformin exerts per se anti-proliferative effects in BC through diverse mechanisms [83], our current findings may suggest the potential usefulness of metformin toward new comprehensive strategies targeting BC.

**Fig. 8:**
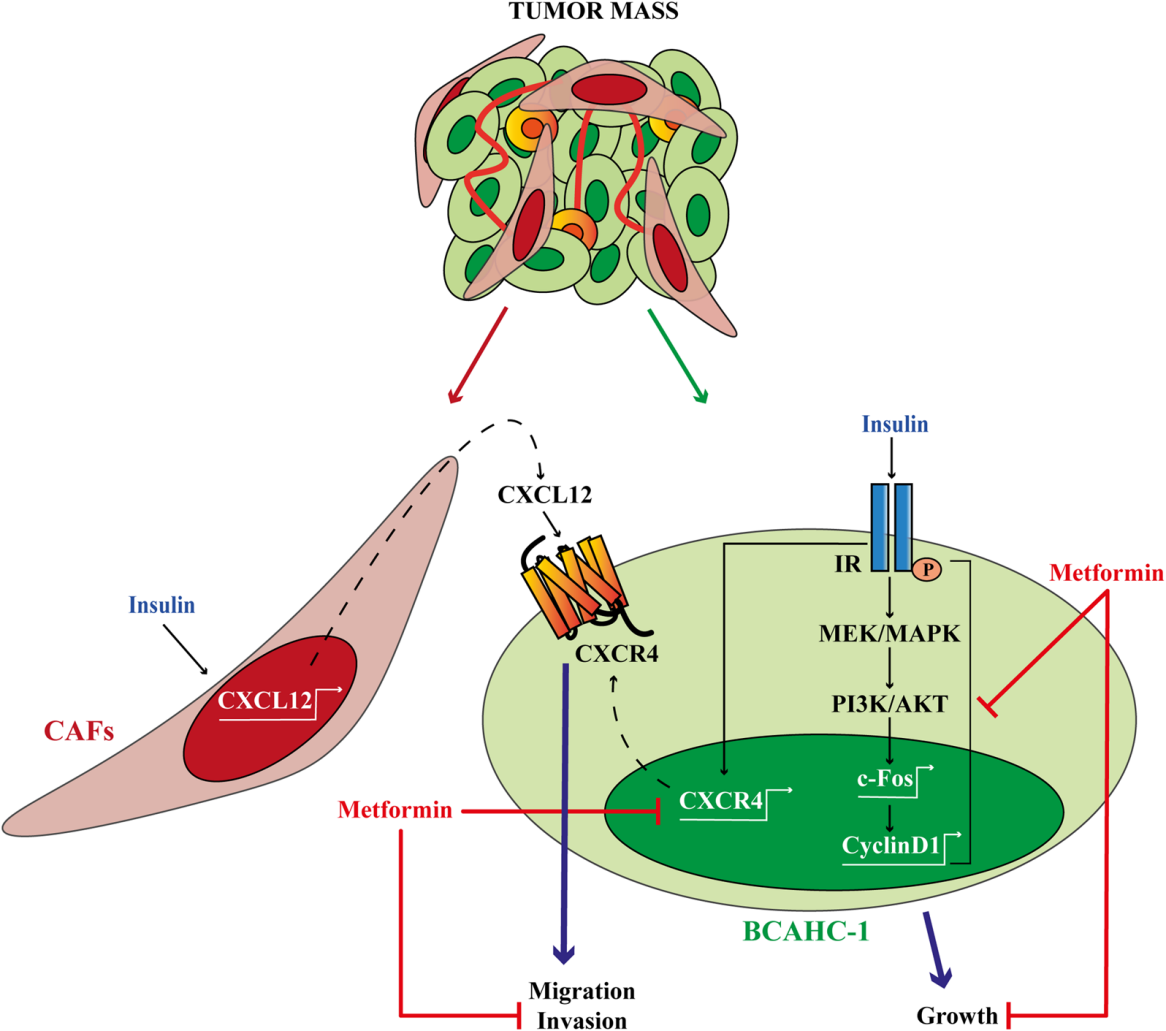
Cartoon depicting metformin action in BCAHC-1 cells. Metformin inhibits the insulin-induced IR, MEK/MAPK and PI3K/AKT activation, c-fos and Cyclin D1 expression as well as proliferative responses in BCAHC-1 cells. Moreover, metformin reduces the insulin-driven feedforward loop linking CXCR4 induction in BCAHC-1 cells to CXCL12 secretion by CAFs, therefore preventing BCAHC-1 cell motility

The tumor microenvironment has been recognized as an important player in BC progression toward the acquisition of aggressive features [100]. CAFs, which are the largest population of stromal cells of BC, influence cancer growth and metastatic spread through the secretion of a variety of hormones, growth factors and inflammatory cytokines [101]. In this vein, a distinctive pro-inflammatory molecule released by CAFs is the chemokine CXCL12, which drives a metastatic phenotype interacting with the cognate receptor CXCR4 [59–64, 102]. CXCR4 is a G protein-coupled chemokine receptor implicated in hemopoietic cell trafficking from bone marrow and lymphoid organs [103, 104]. In addition, emerging data have strengthened the prominent role of CXCR4 in regulating many aspects of BC progression as growth, invasion, angiogenesis, metastasis and resistance to therapies [51, 105]. In particular, the signaling cascade triggered by the CXCL12/CXCR4 axis orchestrates the chemotaxis of BC cells towards CXCL12-enriched tissues, which may therefore arrange the metastatic niche [51, 64, 67, 68, 106–108]. In the present study, CXCR4 emerged as one of the most induced metastatic genes in BCAHC-1 cells exposed to insulin. In line with previous findings showing an association between CXCR4 expression and poor outcome in BC patients [109–112], we performed bioinformatic analysis in ER-positive BC cohorts of patients. Notably, we found that high levels of both CXCR4 and IR are correlated with a worse DFI in ER-positive BC patients. On this basis and considering that a crosstalk between IGF and CXCR4 promotes migratory responses in BC cells [113], we assessed that the up-regulation of CXCR4 expression induced by insulin in BCAHC-1 cells is prevented by metformin. Of note, we also found that insulin triggers CXCL12 induction and release in CAFs. Consistent with the recognized ability of stromal cells to acquire a metastatic secretory phenotype upon insulin exposure [65, 66], we next demonstrated that insulin may drive a feed-forward loop coupling CXCL12 secretion by CAFs to CXCR4 induction in BCAHC-1 cells. Remarkably, we showed that metformin inhibits the acquisition of a migratory and invasive phenotype by BCAHC-1 cells sustained through the aforementioned cooperative network (as schematically depicted in Fig. 8). Nevertheless, further studies are warranted to determine whether metformin may prevent BC motility also via other mechanisms, for instance interfering with recently-identified targets of insulin signaling like the actin filament cross-linking protein filamin-A (FLNA) [114], which has been implicated in the growth and metastatic spread of BC and other hormone-dependent tumors [114–118]. Overall, our data may provide novel insights on the usefulness of metformin in combination therapies targeting the growth effects and motility of the insulin/IR axis in BC.

## Supplementary Information

**Additional file 1: Figure S1.** IR mRNA expression levels (A) and IR-mediated signaling pathway activity (B) among the diverse breast cancer molecular subtypes of the TCGA dataset. Patients were stratified according to the PAM50 gene signature. *p < 0.05, **p < 0.01, ***p < 0.001, ****p < 0.0001.

**Additional file 2: Figure S2.** **A** Proliferation of BCAHC-1 cells after 5 days treatment with vehicle or 10 nM insulin alone or in combination with 1 µM IR inhibitor OSI-906, 1 µM PI3K inhibitor alpelisib or 100 nM MEK inhibitor trametinib. Values of cells treated with vehicle were set as 100% upon which proliferation induced by treatments was determined. **B** Colony formation assay in BCAHC-1 cells exposed to vehicle or 10 nM insulin alone or in combination with 1 µM IR inhibitor OSI-906, 1 µM PI3K inhibitor alpelisib or 100 nM MEK inhibitor trametinib. The plates were stained with Giemsa and colonies were counted following 10 days of incubation (**C**). **D** Representative pictures of spheroids (a single spheroid/well) grown on agar-coated plates upon 20 days treatment with vehicle or 1 µM IR inhibitor OSI-906, 1 µM PI3K inhibitor alpelisib or 100 nM MEK inhibitor trametinib. Scale bar: 100 μm. **E** Quantification of BCAHC-1 spheroid growth. The number of cells treated with vehicle was set as 100% upon which the number of cells upon treatments was determined. Each data point is the mean ± SD of three independent experiments performed in triplicate. (*) indicates significant differences with respect to vehicle sample (p < 0.05); (black square) indicates significant differences with respect to Insulin treated sample (p < 0.05).

## Data Availability

All data generated or analyzed during this study are included in this published article.

## Abbreviations

ACTB: Actin beta
AMPK: Adenosine monophosphate activated protein kinase
AKT: Protein kinase B
BC: Breast cancer
BCA: Bicinchoninic acid
CAFs: Cancer-associated fibroblasts
CM: Conditioned medium
CXCL12: C-X-C Motif Chemokine Ligand 12
CXCR4: CXC chemokine receptor 4
DAPI: 4′,6-Diamidino-2-phenylindole
DFI: Disease-free interval
DMEM/F-12: Dulbecco’s Modified Eagle Medium/Nutrient Mixture F-12
DN/c-Fos: Dominant negative c-Fos
EMT: Epithelial-to-mesenchymal transition
ER: Estrogen receptor
ERα46: 46 kDa ERα splice variant
ERK1/2: Extracellular signal-regulated kinases 1 and 2
FAPα: Fibroblast activation protein alpha
FBS: Fetal bovine serum
FLNA: Filamin-A
GFR: Growth Factor Reduced
IGF-1R: Insulin-like growth factor 1 receptor
IGFs: Insulin-like growth factors
IR: Insulin receptor
IR-A: IR isoform A
IR-B: IR isoform B
ISR1: Insulin receptor substrate 1
MAPK: Mitogen-activated protein kinase
MEK: MAPK/ERK kinase
mTOR: Mechanistic target of rapamycin
ORR: Objective response rate
OS: Overall survival
PBS: Phosphate-buffered saline
PCR: Polymerase chain reaction
PI: Propidium iodide
PI3K: Phosphoinositide 3-kinase
pRL-TK: HSV-thymidine kinase promoter
RSEM: RNA-Seq by expectation–maximization
SDS: Sodium dodecyl sulfate
T2D: Type 2 diabetes
TCGA: The Invasive Breast Cancer Cohort of the Cancer Genome Atlas
