# Supplementary figures and images for: Metformin counteracts stimulatory effects induced by insulin in primary breast cancer cells
Source: J Transl Med. 2022 Jun 7;20:263. doi: 10.1186/s12967-022-03463-y (PMC9172136; doi:10.1186/s12967-022-03463-y)

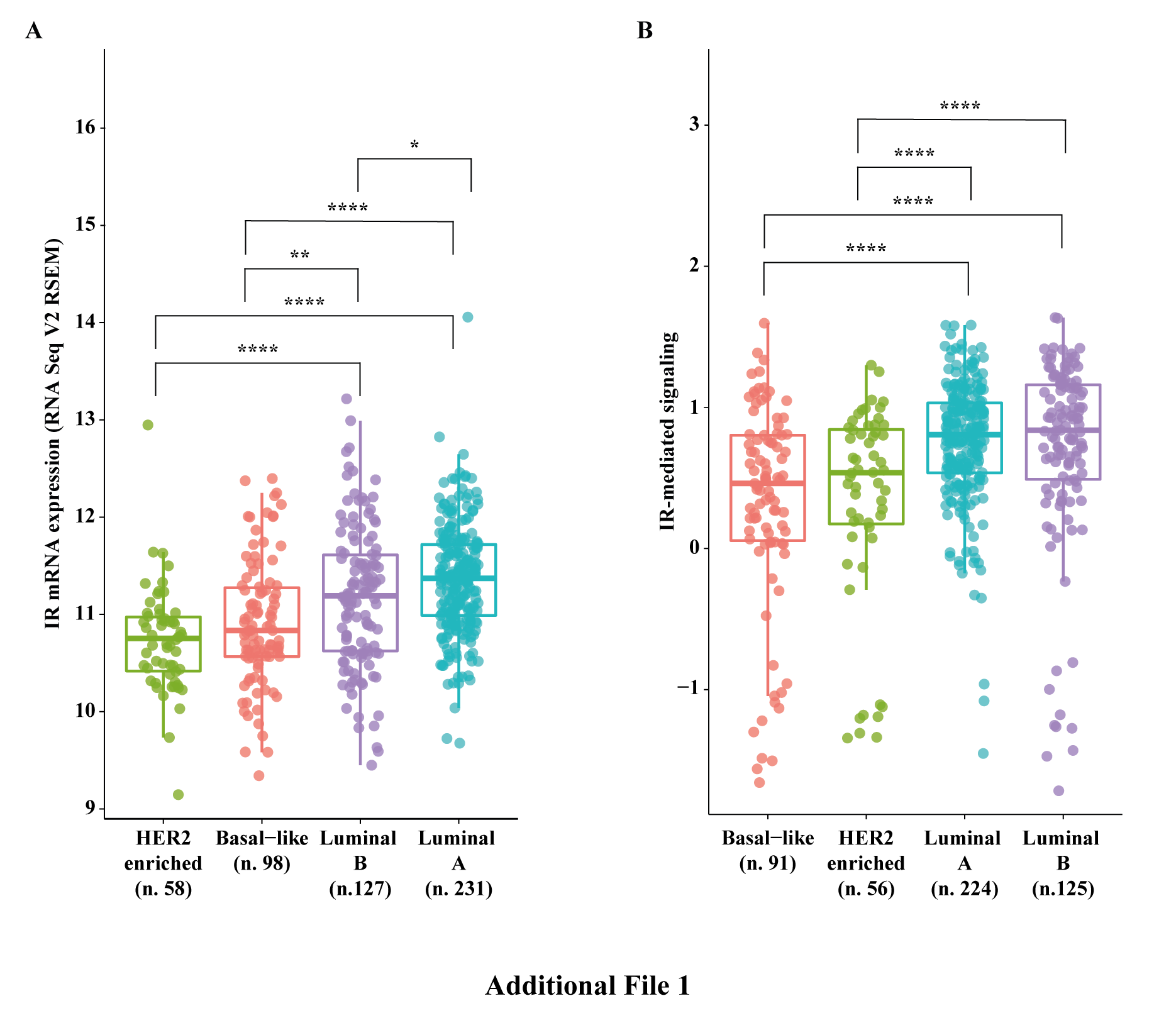

Supplement: Supplementary file 1 — Additional file 1: Figure S1. IR mRNA expression levels (A) and IR-mediated signaling pathway activity (B) among the diverse breast cancer molecular subtypes of the TCGA dataset. Patients were stratified according to the PAM50 gene signature. *p < 0.05, **p < 0.01, ***p < 0.001, ****p < 0.0001. [file 12967_2022_3463_MOESM1_ESM.tif]

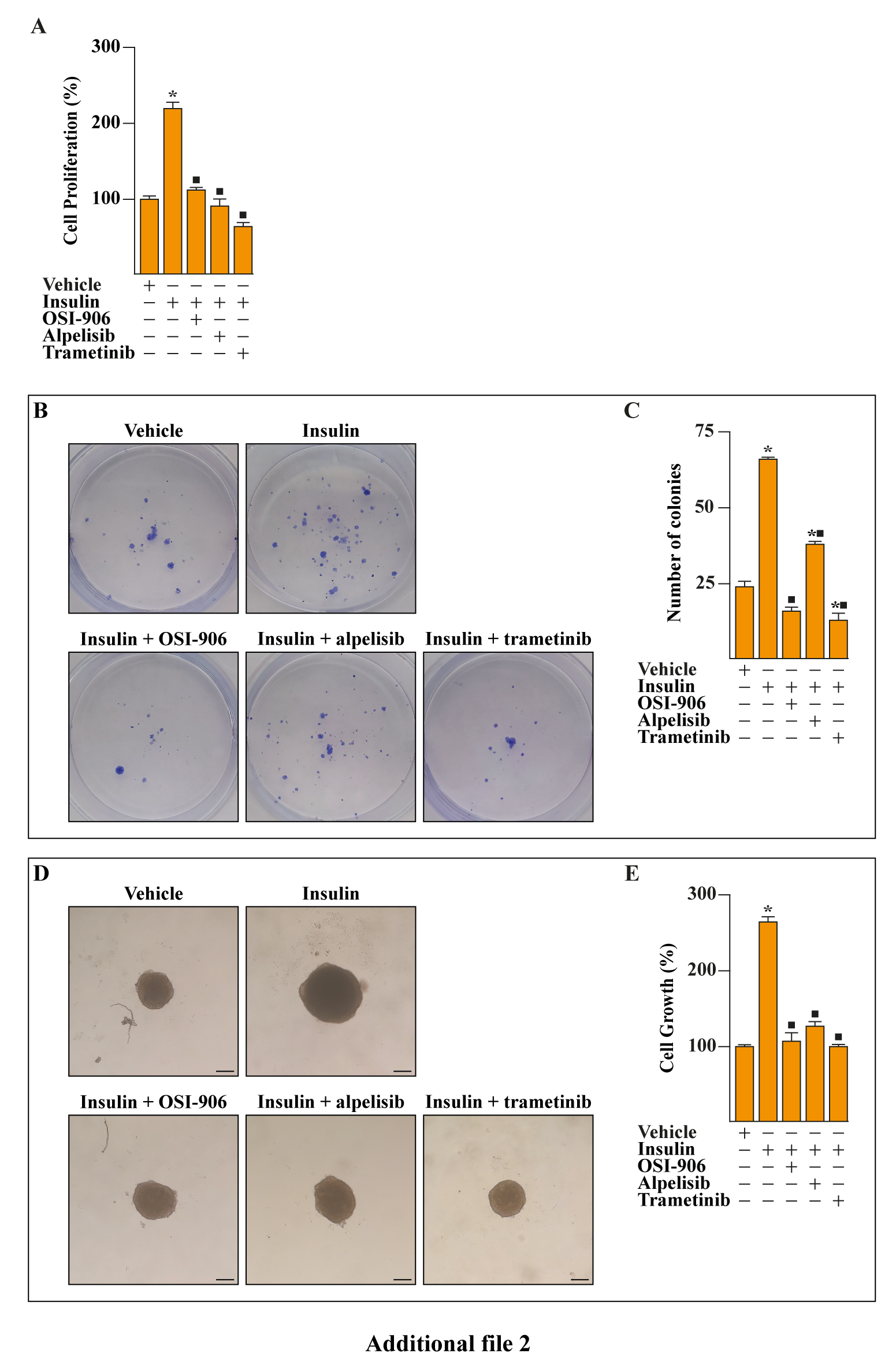

Supplement: Supplementary file 2 — Additional file 2: Figure S2. A Proliferation of BCAHC-1 cells after 5 days treatment with vehicle or 10 nM insulin alone or in combination with 1 µM IR inhibitor OSI-906, 1 µM PI3K inhibitor alpelisib or 100 nM MEK inhibitor trametinib. Values of cells treated with vehicle were set as 100% upon which proliferation induced by treatments was determined. B Colony formation assay in BCAHC-1 cells exposed to vehicle or 10 nM insulin alone or in combination with 1 µM IR inhibitor OSI-906, 1 µM PI3K inhibitor alpelisib or 100 nM MEK inhibitor trametinib. The plates were stained with Giemsa and colonies were counted following 10 days of incubation (C). D Representative pictures of spheroids (a single spheroid/well) grown on agar-coated plates upon 20 days treatment with vehicle or 1 µM IR inhibitor OSI-906, 1 µM PI3K inhibitor alpelisib or 100 nM MEK inhibitor trametinib. Scale bar: 100 μm. E Quantification of BCAHC-1 spheroid growth. The number of cells treated with vehicle was set as 100% upon which the number of cells upon treatments was determined. Each data point is the mean ± SD of three independent experiments performed in triplicate. (*) indicates significant differences with respect to vehicle sample (p < 0.05); (black square) indicates significant differences with respect to Insulin treated sample (p < 0.05). [file 12967_2022_3463_MOESM2_ESM.tif]
